# High Serum Adiponectin Level Is a Risk Factor for Anemia in Japanese Men: A Prospective Observational Study of 1,029 Japanese Subjects

**DOI:** 10.1371/journal.pone.0165511

**Published:** 2016-12-05

**Authors:** Kei Kohno, Hiroto Narimatsu, Yosuke Shiono, Ikuko Suzuki, Yuichi Kato, Ri Sho, Katsumi Otani, Kenichi Ishizawa, Hidetoshi Yamashita, Isao Kubota, Yoshiyuki Ueno, Takeo Kato, Akira Fukao, Takamasa Kayama

**Affiliations:** 1 Department of Neurology, Hematology, Metabolism, Endocrinology and Diabetology, Yamagata University School of Medicine, Yamagata City, Yamagata, Japan; 2 Cancer Prevention and Control Division, Kanagawa Cancer Center Research Institute, Yokohama City, Kanagawa, Japan; 3 Department of Public Health, Yamagata University Graduate School of Medicine, Yamagata City, Yamagata, Japan; 4 Department of Hematology and Cell Therapy, Yamagata University School of Medicine, Yamagata City, Yamagata, Japan; 5 Department of Ophthalmology and Visual Sciences, Yamagata University School of Medicine, Yamagata City, Yamagata, Japan; 6 First Department of Internal Medicine, Yamagata University School of Medicine, Yamagata City, Yamagata, Japan; 7 Second Department of Internal Medicine, Yamagata University School of Medicine, Yamagata City, Yamagata, Japan; 8 Department of Neurosurgery, Yamagata University School of Medicine, Yamagata City, Yamagata, Japan; Universitatsklinikum Freiburg, GERMANY

## Abstract

Erythroid abnormalities including anemia and polycythemia are often observed in the general clinical setting. Because recent studies reported that adiponectin negatively affects hematopoiesis, we performed a prospective observational study to assess the relationship between anemia and adiponectin, as well as other parameters, in 1029 Japanese subjects (477 men and 552 women) 40 years of age and older. Body measurements, blood tests, and nutrition intake studies were performed at baseline, and 5 to 7 years later (follow-up). Hemoglobin (Hb) and hematocrit (Hct) levels in men with high serum adiponectin levels were lower at follow-up than at baseline. Multiple regression analysis showed that age, body mass index, adiponectin, and glutamic-pyruvic transaminase were significantly associated with erythroid-related variables (red blood cells, Hb, and Hct) in both men and women (P <0.05). In a logistic regression analysis, adiponectin, fasting blood glucose, and β-natriuretic peptide were significant risk factors for anemia in men, and blood urea nitrogen and amylase were significant risk factors in women. Physical features and nutrient intake were not risk factors for anemia. Our study demonstrates, both clinically and epidemiologically, that a high serum adiponectin level decreases the amounts of erythroid-related variables and is a risk factor for anemia in Japanese men.

## Introduction

Erythroid abnormalities such as anemia and polycythemia are often encountered in the general clinical setting. Anemia is influenced by aging, as well as iron, the hematopoietic factor erythropoietin (EPO), vitamin B12, folic acid, and other vitamins [1,2]. Some elderly people, however, have anemia that is unrelated to nutrient deficiency, renal insufficiency, or chronic inflammation [3].

Recent studies implicate the cytokine adiponectin in hematopoiesis [4–14]. Adiponectin, which is secreted by adipocytes, enhances insulin sensitivity and has anti-inflammatory and anti-atherosclerosis effects. Its production increases with age and decreases with mast cell enlargement, and loss of adiponectin is a causative factor for diabetes and atherosclerosis [15–18]. Adiponectin negatively regulates the growth of hematopoietic stem cells and myelomonocytes [6–8]; according to some reports, it also regulates bone mass and bone marrow mesenchymal stem cell migration [12–14]. Therefore, it presumably influences erythropoiesis indirectly rather than having a direct effect on erythrocytes. In a small-scale cohort study, high serum adiponectin levels were observed in postmenopausal women with mild anemia [9]. In addition, our previous large-scale cohort study of middle-aged and elderly individuals was the first to show that adiponectin, when highly expressed in serum, decrease red blood cell (RBC) counts in men as well as in women [10]. Recently, Lewerin et al. reported that high adiponectin levels were associated with low blood hemoglobin (Hb) levels in elderly men in a large cohort study [11].

Previous basic biological and clinical epidemiological studies including ours [4–14] suggest that adiponectin is an important factor in hematopoiesis and a potential factor in unexplained anemia. However, because of the cross-sectional design of the clinical epidemiological studies [9–11], it was not clear whether a high adiponectin level was a prognostic factor for anemia and therefore useful in clinical practice. Determination of the relationship between anemia and adiponectin requires a prospective observational study, which has been long awaited.

The Takahata study is a large-scale, population-based study that examines lifestyle habits, collects hematological data and data related to lipid metabolism and diabetes, and conducts investigations involving adiponectin [19,20]. Using the prospective observational data of the Takahata study, we assessed the prognostic value of various factors, most notably adiponectin, in anemia in middle-aged and elderly Japanese people. The factors evaluated were chosen because of their association with anemia and, in addition to adiponectin, include physical features, nutrient uptake parameters, and laboratory test parameters. Our overall goal was to the prognostic impact of adiponectin on the onset of anemia.

## Subjects and Methods

### Subjects

The Takahata cohort study focuses on Japanese people 40 years of age and older. A baseline study of 3519 people (1579 men and 1940 women) was conducted from 2004 to 2006, and a follow-up study of 1029 people (477 men and 552 women) was conducted in 2011. Written informed consent was obtained from all subjects. The Takahata cohort study was approved by the ethics committee of the Yamagata University Faculty of Medicine.

### Laboratory test parameters

The methods used in the baseline study were previously described [10]. The baseline study included body measurements, Brinkman indices, blood tests, assessment of renal and hepatic function, and measurement of lipid, fasting blood glucose (FBG), β-natriuretic peptide (BNP), pancreatic amylase, lipase, cholinesterase (ChE), and serum adiponectin levels. The Japan Diabetes Society index was used to standardize hemoglobin A1c (HbA1c) values and is thought to result in values 0.4% lower than those standardized in accordance with the National Glycohemoglobin Standardization Program. Daily intake of individual nutrients was calculated using the Brief Self-Administered Diet History Questionnaire [21].

The follow-up study included body measurements and the same laboratory tests as in the baseline study with the following exceptions: pancreatic amylase, lipase, and BNP levels and Brinkman indices were not determined, and ChE levels were measured via a different method.

### Statistical analysis

The 1029 patients in the Takahata cohort study were divided into four groups according to the Hb level at baseline as follows: men with anemia (Hb <13.0 g/dL, n = 33); women with anemia (Hb <12.0 g/dL, n = 66); men without anemia (Hb ≥13.0 g/dL, n = 444); and women without anemia (Hb ≥12.0 g/dL, n = 486). In addition, each non-anemia group was subdivided into three subgroups according to serum adiponectin levels at baseline using the k-means clustering method as follows: low (<6.4 μg/mL for men, n = 192 and <9.2 μg/mL for women, n = 219); intermediate (6.4–11.1 μg/mL for men, n = 197 and 9.2–16.0 μg/mL for women, n = 178); and high (>11.1 μg/mL for men, n = 55 and >16.0 μg/mL for women, n = 89). Changes in RBC counts, Hb levels, and hematocrit (Hct) levels (erythroid-related variables) were compared between each group and each subgroup. We performed all analyses on the basis of sex, owing to physiological sex-related differences [1,22]; RBC counts, Hb levels, and Hct levels are significantly higher in men than in women, whereas adiponectin levels are significantly lower.

To identify the baseline variables that affect the erythroid-related variables at follow up, a multiple regression analysis of all subjects was conducted. The dependent variables at follow-up were RBC counts, Hb levels, and Hct levels, and the independent variables at baseline were physical features, nutrient intake, and levels of the laboratory test parameters as follows: serum levels of adiponectin, iron, ferritin, high-sensitivity C-reactive protein (hsCRP), creatinine, glutamic-oxaloacetic transaminase (GOT), glutamic-pyruvic transaminase (GPT), ChE, amylase, lipid parameters, FBG, HbA1c, and BNP.

We also performed a logistic regression analysis in the non-anemic groups to determine whether a high adiponectin was a risk factor for anemia. In the logistic regression analysis, the odds ratios (ORs) of anemia at follow-up were calculated. Multivariate logistic regression models were constructed for adiponectin and the factors found to be significant in univariate logistic regression analyses. All analyses were adjusted for age and body mass index (BMI).

Statistical analyses were performed using R version 2.14.1 software (R Foundation for Statistical Computing, Vienna, Austria) and the EZR software package [23]. All values are presented as mean plus/minus standard deviation. The *t*-test was used to compare mean values between two groups. In the multiple regression analysis, the stepwise backward elimination method based on the P-value was used. In all analyses, P <0.05 was considered significant.

## Results

### Characteristics

Table 1 lists the characteristics of the participants at baseline and follow-up. The follow-up studies occurred 5–7 years after the baseline studies. Mean age at baseline was 62.0 years (range, 40–84 years) for men and 59.6 years (range, 40–83 years) for women. There were significant increases in age and ferritin, hsCRP, FBG, HbA1c, and adiponectin levels, and significant decreases in total protein, albumin, amylase, and low-density lipoprotein cholesterol (LDL-C) levels, in both men and women at follow-up compared with baseline. Also significant were decreases in BMI, diastolic blood pressure, and Hb, serum uric acid, and GPT levels in men at follow-up and increases in systolic blood pressure, RBC counts, and Hct and GOT levels in women at follow-up. No patients had polycythemia (Hb >18.5 g/dL in men, Hb >16.5 g/dL in women).

**Table 1:** Characteristics of the participants in the baseline and follow-up studies.

Factor	Baseline study		Follow-up study	
	Mean ± SD		Mean ± SD	
	Men (n = 477)	Women (n = 552)	Men (n = 477)	Women (n = 552)
Age (years)	62.0 ± 9.2	59.6 ± 8.9	68.6 ± 9.3*	66.2 ± 9.1*
BMI (kg/m^2^)	23.5 ± 2.6	23.2 ± 3.2	23.4 ± 2.8*	23.1 ± 3.4
Systolic BP (mmHg)	135.5 ± 15.0	130.4 ± 15.1	136.8 ± 17.4	132.3 ± 16.4*
Diastolic BP (mmHg)	81.7 ± 9.6	76.8 ± 10.0	79.8 ± 10.5*	76.2 ± 9.2
Brinkman index	395 ± 390	13 ± 63		
RBC (10^4^/mm^3^)	462.0 ± 39.2	427.3 ± 32.8	463.7 ± 43.9	433.8 ± 33.3*
Hb (g/dL)	14.6 ± 1.1	13.0 ± 1.0	14.4 ± 1.3*	13.0 ± 0.9
Hct (%)	43.6 ± 3.2	39.1 ± 2.8	43.6 ± 3.6	40.3 ± 2.7*
Fe (μg/dL)	112.4 ± 37.3	101.1 ± 31.0	110.5 ± 39.4	102.2 ± 30.9
Ferritin (ng/mL)	102.0 ± 94.5	55.6 ± 44.0	121.8 ± 106.1*	69.9 ± 53.1*
hsCRP (ng/mL)	1286 ± 2846	663 ± 1679	1402 ± 4213*	736 ± 1367*
TP (g/dL)	7.48 ± 0.47	7.47 ± 0.38	7.40 ± 0.48*	7.41 ± 0.47*
ALB (g/dL)	4.53 ± 0.27	4.49 ± 0.23	4.44 ± 0.30*	4.46 ± 0.28*
BUN (mg/dL)	16.8 ± 4.6	15.5 ± 3.9	17.1 ± 4.9	15.9 ± 3.8
Cr (mg/dL)	0.78 ± 0.33	0.58 ± 0.09	0.81 ± 0.43	0.58 ± 0.11
Serum uric acid (mg/dL)	5.7 ± 1.3	4.4 ± 0.9	5.6 ± 1.2*	4.4 ± 1.0
GOT (IU/L)	26.3 ± 9.2	22.5 ± 7.0	26.8 ± 8.8	23.8 ± 6.8*
GPT (IU/L)	25.3 ± 13.2	20.8 ± 11.2	23.4 ± 11.3*	20.3 ± 10.4
γGTP (IU/L)	48.9 ± 56.6	24.9 ± 18.7	47.2 ± 66.1	24.3 ± 20.0
ChE (U/L) ^※^	5361 ± 1002	5518 ± 999	327.9 ± 69.8	350.5 ± 64.6
Amylase (U/L)	108.8 ± 65.8	108.6 ± 31.6	83.8 ± 27.2*	82.2 ± 27.5*
Pancreatic amylase (U/L)	29.3 ± 9.0	31.1 ± 8.6		
Lipase (U/L)	32.1 ± 10.5	32.9 ± 10.3		
TC (mg/dL)	193.1 ± 30.4	208.0 ± 30.5	193.0 ± 32.6	207.9 ± 32.5
HDL-C (mg/dL)	55.7 ± 13.7	62.6 ± 14.3	55.8 ± 14.5	62.3 ± 14.0
LDL-C (mg/dL)	118.7 ± 28.2	129.3 ± 29.6	112.7 ± 28.3*	122.5 ± 28.6*
TG (mg/dL)	117.1 ± 80.5	94.5 ± 46.3	120.6 ± 75.7	97.0 ± 44.7
FBG (mg/dL)	96.8 ± 18.1	90.3 ± 9.7	99.4 ± 18.1*	93.5 ± 15.0*
HbA1c (%)	5.2 ± 0.6	5.1 ± 0.4	5.4 ± 0.5*	5.3 ± 0.4*
BNP (pg/mL)	24.8 ± 29.4	26.9 ± 22.9		
Adiponectin (μg/mL)	7.5 ± 3.6	11.2 ± 5.4	11.7 ± 6.8*	15.7 ± 8.2*
Total Caloric Intake (kcal/day)	2432 ± 681	2123 ± 557		
Protein (g/day)	77.2 ± 29.3	76.8 ± 28.3		
Animal Protein (g/day)	37.8 ± 22.2	39.8 ± 21.5		
Vegetable Protein (g/day)	39.4 ± 11.1	37.0 ± 10.8		
Fat (g/day)	55.5 ± 22.2	59.4 ± 21.2		
Animal Fat (g/day)	22.2 ± 12.3	24.2± 11.9		
Vegetable Fat (g/day)	33.2 ± 12.5	35.2 ± 12.4		
Carbohydrates (g/day)	348.4 ± 99.1	310.1 ± 79.0		
Sodium (mg/day)	5217 ± 1527	5045 ± 1512		
Potassium (mg/day)	2638 ± 1099	2878 ± 1071		
Calcium (mg/day)	598.5 ± 270.9	646.3 ± 265.9		
Magnesium (mg/day)	295.9 ± 105.0	298.2 ± 102.0		
Phosphorus (mg/day)	1164 ± 446	1169 ± 429		
Iron (mg/day)	9.09 ± 3.46	9.55 ± 3.43		
Zinc (mg/day)	9.67 ± 3.06	9.37 ± 2.84		
Copper (mg/day)	1.50 ± 0.44	1.44 ± 0.42		
Manganese (mg/day)	4.46 ± 1.32	4.17 ± 1.14		
Retinol Equivalent (μg/day)	755.7 ± 633.3	789.9 ± 531.6		
Vitamin B1 (mg/day)	0.78 ± 0.31	0.85 ± 0.30		
Vitamin B2 (mg/day)	1.43 ± 0.55	1.50 ± 0.52		
Vitamin B6 (mg/day)	1.37 ± 0.59	1.38 ± 0.55		
Vitamin B12 (μg/day)	10.6 ± 7.5	10.7 ± 7.3		
Folic Acid (μg/day)	378.6 ± 163.3	410.6 ± 162.3		
Vitamin C (mg/day)	106.4 ± 57.5	129.4 ± 60.4		
Vitamin D (μg/day)	15.7 ± 12.3	16.5 ± 12.3		
Vitamin K (mg/day)	459.3 ± 232.3	504.3 ± 233.5		
Cholesterol (mg/day)	322.9 ± 174.3	335.3 ± 159.4		
Salt (g/day)	13.1 ± 3.8	12.7 ± 3.8		
Sucrose (g/day)	20.6 ± 8.4	22.6 ± 9.3		
Alcohol (g/day)	26.9 ± 29.6	2.1 ± 7.6		

* Significant difference (P <0.05) between the baseline and follow-up values.

^※^ChE levels were measured via different methods in the baseline and follow-up studies.

SD, standard deviation; BMI, body mass index; BP, blood pressure; RBC, red blood cell; Hb, hemoglobin; Hct, hematocrit; Fe, serum iron; hsCRP, high-sensitivity C-reactive protein; TP, total protein; ALB, albumin; BUN, blood urea nitrogen; Cr, creatinine; GOT, glutamic-oxaloacetic transaminase; GPT, glutamic-pyruvic transaminase; ChE, cholinesterase; TC, total cholesterol; HDL-C, high-density lipoprotein cholesterol; LDL-C, low-density lipoprotein cholesterol; TG, triglycerides; FBG, fasting blood glucose; HbA1c, hemoglobin A1c; BNP, β-natriuretic peptide.

### Levels of the erythroid-related variables as a function of time

**Fig 1:**
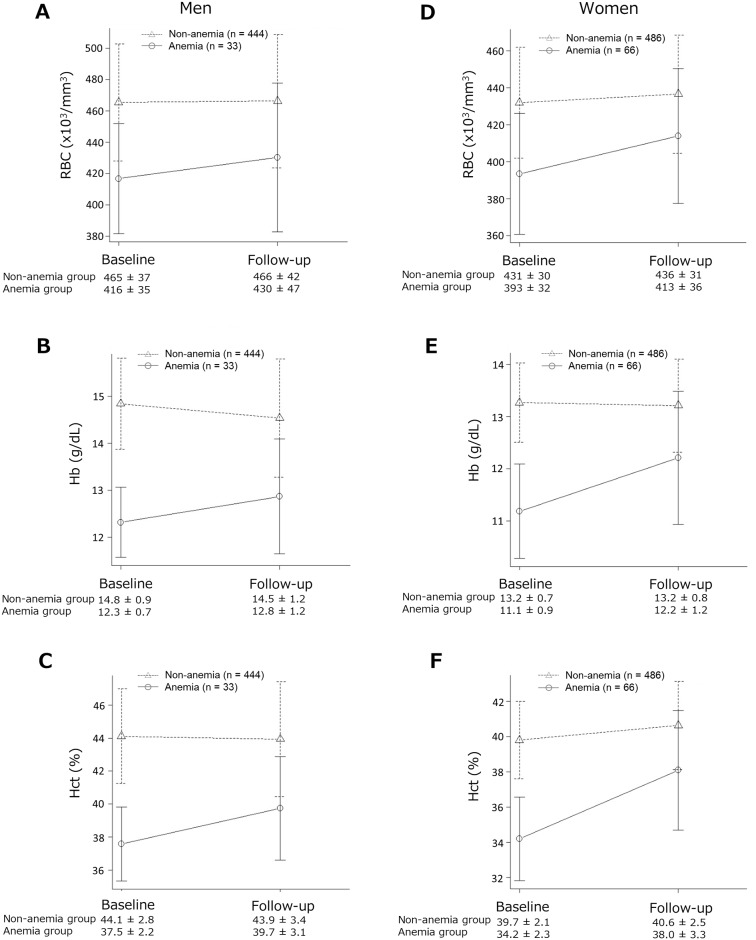
Differences in erythroid parameters stratified according to hemoglobin (Hb) levels at baseline. In men without anemia, Hb [Fig 1.B] and hematocrit (Hct) [Fig 1.C] levels were lower at follow-up than at baseline, whereas red blood cells (RBC) counts [Fig 1.A] were unchanged. In women without anemia, RBC counts [Fig 1.D] and Hct levels [Fig 1.F] were higher at follow-up than at baseline, whereas Hb levels [Fig 1.E] were unchanged. In both men and women with anemia, all three parameters were higher at follow-up.

Fig 1 shows the Hb and Hct levels and RBC counts (the erythroid-related variables) at baseline and follow-up. In anemic men and women, the RBC counts and levels of Hb and Hct were higher at follow-up than at baseline. Conversely, in non-anemic men, Hb and Hct levels were lower at follow-up than at baseline, whereas only Hb levels were unchanged in non-anemic women.

**Fig 2:**
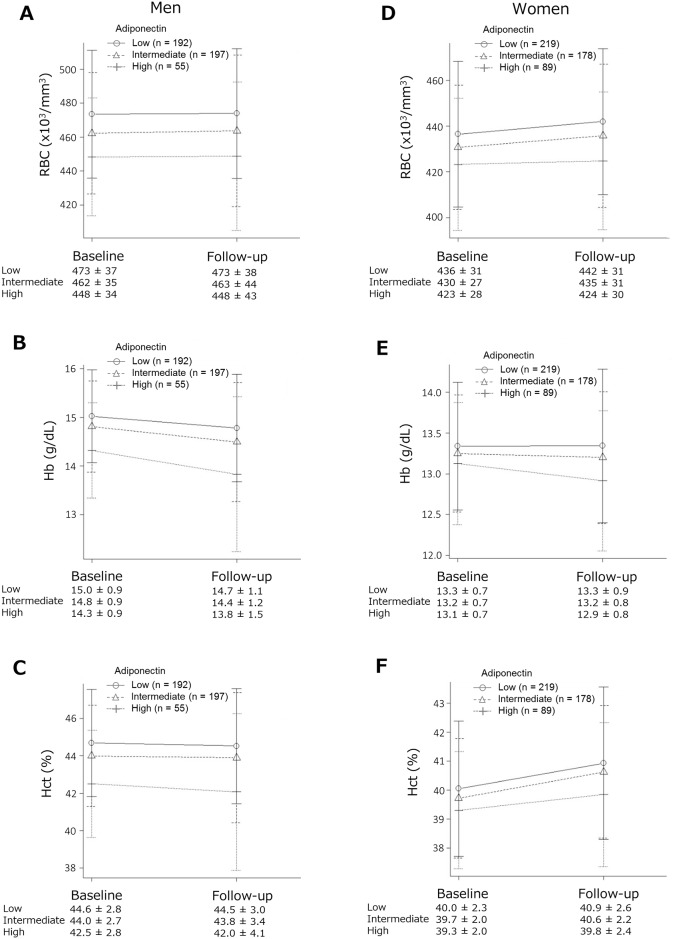
Differences in the levels of the erythroid-related parameters stratified according to adiponectin levels at baseline. The non-anemia groups were subdivided into three subgroups (low, intermediate, and high) according to serum adiponectin levels at baseline. In men in the high adiponectin subgroup, hemoglobin (Hb) [Fig 2.B] and hematocrit (Hct) [Fig 2.C] levels were lower at follow-up than at baseline, whereas red blood cell (RBC) counts [Fig 2.A] were unchanged. In women in the high adiponectin subgroup, Hb levels [Fig 2.E] were lower, and RBC counts [Fig 2.D] and Hct [Fig 2.F] were higher at follow-up than at baseline.

Fig 2 shows the values of the erythroid-related variables stratified according to adiponectin levels. In the non-anemia groups, Hb and Hct levels and RBC counts were lower in the high adiponectin subgroup than in the intermediate and low subgroups at baseline. In men with high adiponectin levels, Hb and Hct levels were lower at follow-up than at baseline, whereas RBC counts were unchanged. In women with high adiponectin levels, Hb levels were lower at follow-up, whereas RBC counts and Hct levels were higher.

### Multiple regression analysis of the erythroid-related variables

Tables 2 and 3 show the results of a multiple regression analysis of the erythroid-related variables at follow-up for each independent variable. The physical variables significantly associated with the erythroid-related variables were age and BMI in both sexes. The laboratory variables significantly associated with the erythroid-related variables excepting RBC counts in women were adiponectin and GPT levels. Creatinine levels in men and LDL-C levels in women were also significantly associated with the erythroid-related variables. Nutrient intake was not a significant factor in men, whereas some nutrient intake-related variables were significant in women. However, the R^2^ value was low (Table 3), and the P-values for Hb (P = 0.068) and Hct (P = 0.099) were not significant. In summary, the baseline factors that affected the erythroid-related variables at follow-up were age and BMI in both sexes, and the effects of laboratory and nutrient intake factors were sex-dependent.

**Table 2:** Results of the multiple regression analysis in men.

RBC at follow-up			Hb at follow-up			Hct at follow-up		
**Model 1**			**Model 1**			**Model 1**		
Adjusted R2	0.1517		Adjusted R2	0.1296		Adjusted R2	0.1179	
Variable	r	P	Variable	r	P	Variable	r	P
(Intercept)	515.3		(Intercept)	14.13		(Intercept)	44.75	
**Age**	-1.725	<0.001	**Age**	-0.043	<0.001	**Age**	-0.112	<0.001
**BMI**	2.326	<0.001	**BMI**	0.077	<0.001	**BMI**	0.230	<0.001
			Diastolic BP	0.012	0.044	Brinkman index	0.001	0.007
			Brinkman index	0.0004	0.006			
**Model 2**			**Model 2**			**Model 2**		
Adjusted R2	0.1114		Adjusted R2	0.07406		Adjusted R2	0.0581	
Variable	r	P	Variable	r	P	Variable	r	P
(Intercept)	571.3		(Intercept)	17.1		(Intercept)	50.45	
Age	-1.734	<0.001	Age	-0.043	<0.001	Age	-0.109	<0.001
**Model 3**			**Model 3**			**Model 3**		
Adjusted R2	0.2095		Adjusted R2	0.1956		Adjusted R2	0.1645	
Variable	r	P	Variable	r	P	Variable	r	P
(Intercept)	572.7		(Intercept)	17.28		(Intercept)	49.16	
**Age**	-1.222	<0.001	**Age**	-0.025	<0.001	**Age**	-0.067	<0.001
**Adiponectin**	-2.058	<0.001	**Adiponectin**	-0.081	<0.001	**Adiponectin**	-0.207	<0.001
**Cr**	-12.46	0.026	**Cr**	-0.426	0.010	**Cr**	-1.039	0.026
GOT	-0.865	<0.001	**GPT**	0.018	<0.001	**GPT**	0.041	0.001
**GPT**	0.734	<0.001	ChE	0.0001	0.024	ChE	0.0003	0.032
BNP	-0.143	0.032	Amylase	-0.001	0.041	FBG	-0.019	0.034
			HbA1c	-0.248	0.004			

Model 1 was adjusted for age and physical features; model 2 was adjusted for age and nutrient intake; model 3 was adjusted for age and levels of the laboratory test parameters at baseline.

r, regression coefficient; P, P-value. RBC, red blood cell; Hb, hemoglobin; Hct, hematocrit; BMI, body mass index; BP, blood pressure; Cr, creatinine; GOT, glutamic-oxaloacetic transaminase; GPT, glutamic-pyruvic transaminase; BNP, β-natriuretic peptide; ChE, cholinesterase; HbA1c, hemoglobin A1c; FBG, fasting blood glucose.

**Table 3:** Results of the multiple regression analysis in women.

RBC at follow-up			Hb at follow-up			Hct at follow-up		
**Model 1**			**Model 1**			**Model 1**		
Adjusted R2	0.09897		Adjusted R2	0.03185		Adjusted R2	0.02946	
Variable	r	P	Variable	r	P	Variable	r	P
(Intercept)	414.9		(Intercept)	12.74		(Intercept)	38.00	
**Age**	-0.948	<0.001	**Age**	-0.013	0.004	**Age**	-0.035	0.006
**BMI**	1.480	<0.001	**BMI**	0.049	<0.001	**BMI**	0.096	0.010
Diastolic BP	0.533	<0.001				Diastolic BP	0.028	0.019
**Model 2**			**Model 2**			**Model 2**		
Adjusted R2	0.04521		Adjusted R2	0.0231		Adjusted R2	0.01951	
Variable	r	P	Variable	r	P	Variable	r	P
(Intercept)	481.8		(Intercept)	13.94		(Intercept)	50.45	
**Age**	-0.737	<0.001	**Age**	-0.012	0.014	**Age**	-0.109	<0.001
**Total caloric intake**	0.091	0.010	**Total caloric intake**	0.007	0.003	**Total caloric intake**	0.020	0.004
**Alcohol**	-0.919	0.004	**Alcohol**	-0.045	0.017	**Alcohol**	-0.130	0.014
**Animal protein**	-1.003	0.014	**Animal protein**	-0.041	0.001	**Animal protein**	-0.108	0.002
**Vegetable fat**	-1.076	0.010	Animal fat	-0.055	0.017	Animal fat	-0.156	0.015
**Carbohydrates**	-0.382	0.017	**Vegetable fat**	-0.086	0.001	**Vegetable fat**	-0.235	0.001
Iron	-4.423	0.044	**Carbohydrates**	-0.034	0.002	**Carbohydrates**	-0.091	0.004
Vitamin B12	1.138	0.043	**Folic acid**	0.001	0.002	**Folic acid**	0.003	0.005
**Folic acid**	0.075	0.015						
**Model 3**			**Model 3**			**Model 3**		
Adjusted R2	0.1204		Adjusted R2	0.08669		Adjusted R2	0.058	
Variable	r	P	Variable	r	P	Variable	r	P
(Intercept)	454.3		(Intercept)	13.94		(Intercept)	44.70	
**Age**	-0.903	<0.001	**Age**	-0.011	0.013	**Age**	-0.029	0.025
ChE	0.004	0.002	Adiponectin	-0.025	0.001	Adiponectin	-0.049	0.027
Amylase	-0.125	0.003	**GPT**	0.011	0.003	**GPT**	0.026	0.014
**LDL-C**	0.112	0.019	TP	-0.242	0.032	ChE	0.0003	0.023
TG	0.083	0.007	HDL-C	0.007	0.018	TP	-0.760	0.018
			**LDL-C**	0.004	0.002	**LDL-C**	0.010	0.008
			TG	0.002	0.009			
			Fe	-0.248	0.004			

Model 1 was adjusted for age and physical features; model 2 was adjusted for age and nutrient intake; model 3 was adjusted for age and levels of the laboratory test parameters at baseline.

r, regression coefficient; P, P-value. RBC, red blood cell; Hb, hemoglobin; Hct, hematocrit; BMI, body mass index; BP, blood pressure; ChE, cholinesterase; LDL-C, low-density lipoprotein cholesterol; TG, triglycerides; GPT, glutamic-pyruvic transaminase; TP, total protein; HDL-C, high-density lipoprotein cholesterol; Fe, serum iron.

### Risk factors for anemia

We assessed the potential risk factors for anemia in non-anemic men (n = 444) and women (n = 486) at baseline. At follow-up, 33 (7.4%) men and 33 (6.7%) women were anemic. The mean Hb levels at follow-up were 11.5 g/dL (range, 9–12.9 g/dL) for men and 11.3 g/dL (range, 8.2–11.9 g/dL) for women. The degree of anemia was mild to moderate.

In a logistic regression analysis where the independent variables were physical features, age (OR, 1.075; P = 0.001) was the only significant risk factor for anemia in men. In the logistic regression analysis where the independent variables were related to nutrient intake, there was no significant risk factor for anemia (P >0.05 for all variables).

Table 4 shows the results of the logistic regression analysis where the independent variables were laboratory test parameters. In the univariate analysis, the significant laboratory test parameters for men were adiponectin, blood urea nitrogen (BUN), ChE, high-density lipoprotein cholesterol, FBG, and BNP. In the multivariate analysis, the significant factors for men were adiponectin (OR, 1.116; P = 0.033), FBG (OR, 1.018; P = 0.029), and BNP (OR, 1.010; P = 0.045). Mean age and serum adiponectin, FBG and BNP levels at baseline were significantly higher in men with anemia (n = 33) than in men without anemia (n = 411) at follow-up (age, 66.7 vs. 61.2 years, P <0.01; adiponectin, 9.9 vs. 7.1 μg/mL, P <0.01; FBG, 103.4 vs. 96.3 mg/dL, P = 0.02; BNP, 18.4 vs. 16.5 pg/mL, P <0.01). In women, BUN (OR, 0.862; P = 0.007) and amylase (OR, 1.013; P = 0.009) were significant risk factors in both the univariate and multivariate analyses. Mean BUN levels were significantly lower and serum amylase levels were significantly higher in women with anemia (n = 33) than in women without anemia (n = 453) (BUN, 14.0 vs. 15.7 mg/dL, P = 0.02; amylase, 122.0 vs. 107.3 U/L, P = 0.01). There were no significant differences in adiponectin levels in anemic versus non-anemic women (12.2 vs. 10.9 μg/mL, P = 0.19). High serum adiponectin levels were a risk factor for anemia in men but not in women.

**Table 4:** Risk factors for anemia based on odds ratios.

**[Men]**							
		Univariate analysis			Multivariate analysis		
Variable		Odds ratio	95% CI	P-value	Odds ratio	95% CI	P-value
Age	(linear by year)	1.075	1.028–1.123	0.001			
BMI	(linear by 1 kg/m^2^)	0.917	0.806–1.052	0.224			
**Adiponectin**	(linear by 1 μg/mL)	1.199	1.101–1.303	<0.001	1.190	1.089–1.300	**<0.001**
BUN	(linear by 1 mg/dL)	1.109	1.023–1.203	0.011			
ChE	(linear by 1 U/L)	1.000	1.000–1.000	0.011			
HDL-C	(linear by 1 mg/dL)	1.025	1.001–1.051	0.042			
**FBG**	(linear by 1mg/dL)	1.016	1.001–1.031	0.036	1.021	1.006–1.038	**0.007**
**BNP**	(linear by 1pg/mL)	1.012	1.004–1.020	0.004	1.011	1.002–1.019	**0.012**
**[Women]**							
		Univariate analysis			Multivariate analysis		
Variable		Odds ratio	95% CI	P-value	Odds ratio	95% CI	P-value
**Age**	(linear by year)	1.036	0.990–1.081	0.103	1.049	1.005–1.095	**0.027**
BMI	(linear by 1 kg/m^2^)	0.961	0.854–1.071	0.452			
Adiponectin	(linear by 1 μg/mL)	1.039	0.980–1.102	0.195			
**BUN**	(linear by 1 mg/dL)	0.892	0.800–0.990	0.025	0.862	0.769–0.961	**0.007**
**Amylase**	(linear by 1 U/L)	1.012	1.003–1.021	0.012	1.013	1.003–1.023	**0.009**

CI, confidence level; BMI, body mass index; BUN, blood urea nitrogen; ChE, cholinesterase; HDL-C, high-density lipoprotein cholesterol; FBG, fasting blood glucose; BNP, β-natriuretic peptide.

## Discussion

Our prospective observational study demonstrates that high adiponectin levels decrease the levels of three erythroid-related variables (Hb levels, Hct levels, and RBC counts). In Japanese men, a high adiponectin level was a risk factor for anemia. In Japanese women, a high adiponectin level reduced the levels of the erythroid-related variables but was not a risk factor for anemia. Basic biological studies suggest that adiponectin negatively regulates the growth of hematopoietic cells [6,7], and our study clinically and epidemiologically shows that adiponectin negatively affects erythropoiesis.

The present study indicates that Japanese men with high adiponectin levels will develop anemia. It also provides relevant information for optimal management of anemia. A previous study reported that plasma adiponectin levels correlated negatively with body fat percentage in older men [22], and our study showed that serum adiponectin levels correlated negatively with BMI (S1 Fig). Reduction of high adiponectin levels in men requires intervention to prevent the development of anemia. However, low adiponectin levels can increase the risk of cardiac disease and diabetes [17,18]; thus, careful clinical management is needed. Although adiponectin production increases with age, the normal values for each age have not been determined. A future goal is to establish appropriate serum adiponectin levels for each age.

None of subjects in this study had severe anemia (Hb <8 mg/dL in men, Hb <7 mg/dL in women); instead they had mild to moderate anemia. Several studies have associated high adiponectin levels with mild anemia [9–11]. If anemia is severe, factors in addition to adiponectin (e.g., hemorrhage and hematologic malignancies) should be considered.

In our previous cross-sectional study [10], we suggested that management of anemia according to sex is necessary, and this study confirms this premise. In this study, the risk factors for anemia varied according to sex. The risk factors for men were high FBG and BNP levels. Anemia is a well-known complication of diabetes [24–26]. Shola and Olugbenda found that a hyperglycemic environment caused anemia in diabetic rats [27], perhaps by eliciting an intravascular hemolytic event. BNP is a cardiac hormone secreted from the ventricles, and plasma BNP is a biomarker of ventricular dysfunction. Several studies have shown that patients with anemia have high BNP levels, thereby suggesting that elevated BNP levels are potential indicators of anemia and are associated with low Hb levels in the absence of heart failure [28–30]. The risk factors for anemia in women in our study were low BUN levels and high serum amylase levels. It has been anecdotally reported that BUN levels correlate with protein intake [31,32] and that thin people have high serum amylase levels [33]. In agreement, we found that BUN levels were significantly associated with protein intake and that serum amylase levels were significantly associated with BMI in women (S2 Fig). Collectively, the above findings suggest that sex-based management of parameters such as cardiac function, carbohydrate metabolism, and nutritional status could prevent the onset of anemia.

The present study provides new information about the relationship between anemia and risk factors. However, it has several limitations. First, intervention was not controlled, and the possibility that intervention may have affected the results in some patients cannot be ruled out. Some but not all participants received health education at baseline. Although some patients had increased levels of all three erythroid-related variables at follow-up, they were not anemic. This may be due to routine medical examinations, a high health consciousness, and a healthy lifestyle owing to health education in these patients.

Second, our cohort did not include people less than 40 years of age. Because the number of premenopausal women in our cohort was small, we did not consider menstrual status. However, our previous study showed that menstrual status strongly influenced the development of anemia, whereas lifestyle and eating habits had little effect in women [10]. Thus, whether adiponectin contributes to anemia in younger adults, including premenopausal women, was not addressed. A prospective observational study in young people is warranted.

Third, we did not examine other factors related to anemia, most notably EPO. A recent cross-sectional study suggests that adiponectin affects Hb levels along with EPO, estradiol, and thyroid hormone [11]. In our study, we measured serum iron, ferritin, creatinine, and hsCRP levels but not EPO, estradiol, or thyroid hormone levels. hsCRP levels were within the normal range in all subjects, and only three mem (n = 444, 0.6%) had creatinine levels >1.2 mg/dL (range, 1.3–1.4 mg/dL). These findings suggest that these factors are unrelated to renal or inflammatory anemia. Sixteen men (n = 202, 7.9%) and 11 women (n = 222, 4.9%) had decreased serum ferritin levels (<20 ng/mL in men, <10 ng/mL in women). The three men and one woman with low ferritin levels became anemic. The Cochran-Armitage test (a trend analysis) showed no significant correlation between low ferritin levels and anemia (data not shown). Future studies that include EPO and other hormones are necessary.

In conclusion, this clinical cohort study shows that high adiponectin levels significantly correlate with anemia in Japanese men. It provides important evidence for determining the basic mechanism whereby adiponectin prevent anemia.

## Supporting Information

S1 Fig Correlation of adiponectin levels and body mass index (BMI) in men. Serum adiponectin levels correlated negatively with BMI in men.
(TIF)

S2 Fig Correlation of blood urea nitrogen (BUN) levels and protein intake, amylase levels and body mass index (BMI) in women. BUN levels directly correlated protein intake (A) and serum amylase levels inversely correlated with BMI (B) in women.
(TIF)
