# Supplementary figures and images for: High Serum Adiponectin Level Is a Risk Factor for Anemia in Japanese Men: A Prospective Observational Study of 1,029 Japanese Subjects
Source: PLoS One. 2016 Dec 5;11(12):e0165511. doi: 10.1371/journal.pone.0165511 (PMC5137881; doi:10.1371/journal.pone.0165511)

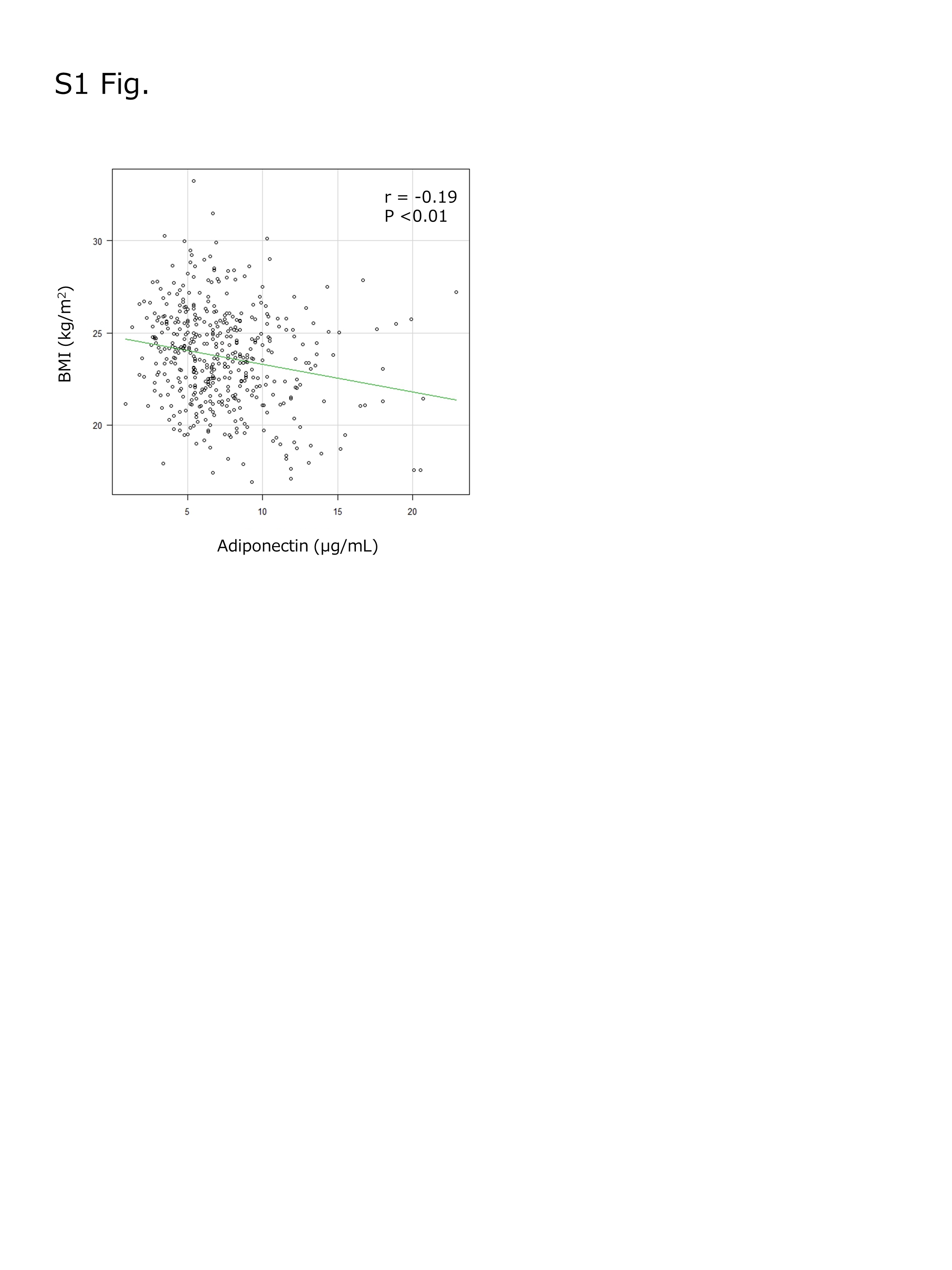

Supplement: S1 Fig — Serum adiponectin levels correlated negatively with BMI in men. (TIF) [file pone.0165511.s001.TIF]

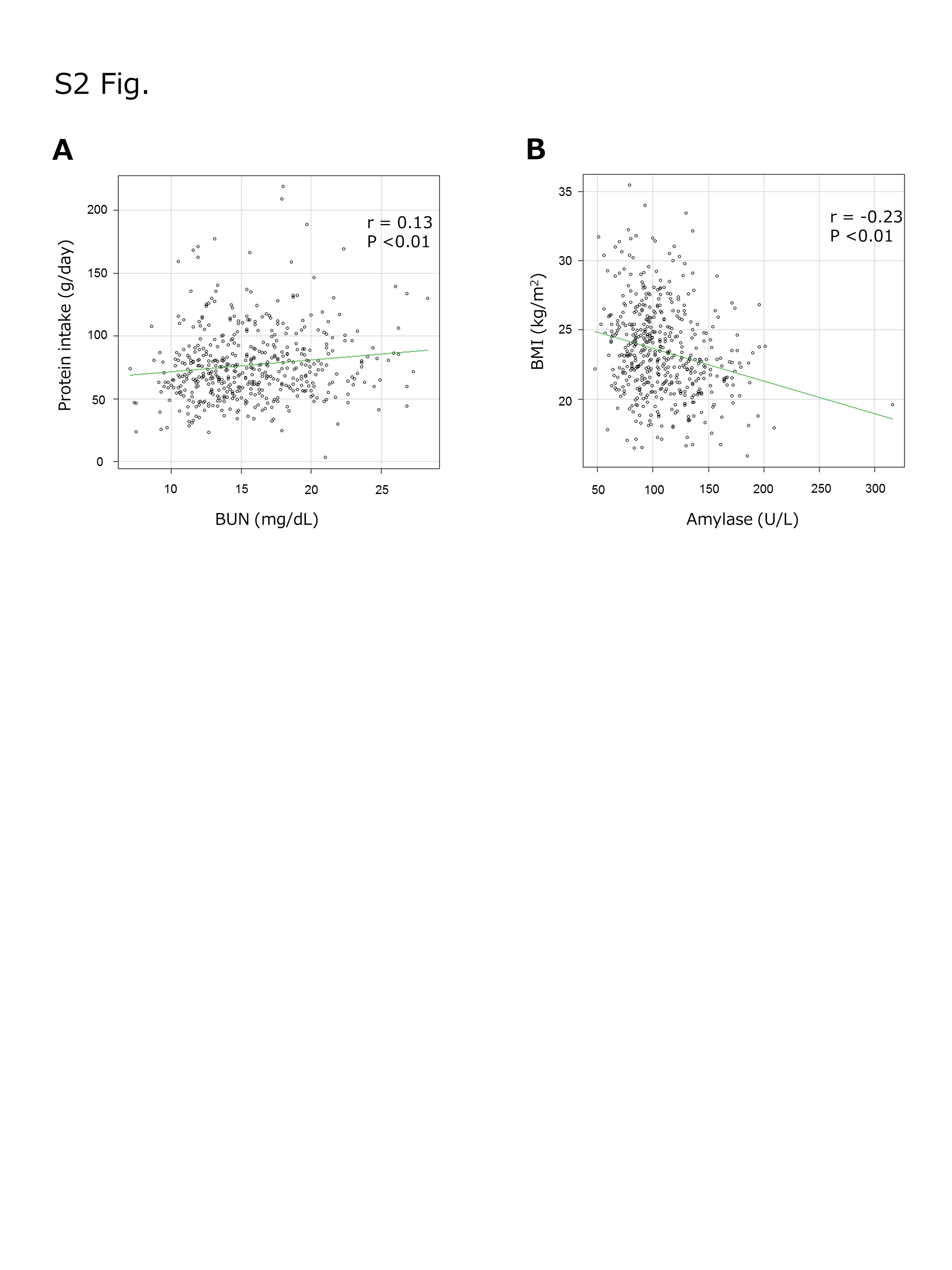

Supplement: S2 Fig — BUN levels directly correlated protein intake (A) and serum amylase levels inversely correlated with BMI (B) in women. (TIF) [file pone.0165511.s002.TIF]
